# Unravelling Trait–Environment Relationships at Local and Regional Scales in Temperate Forests

**DOI:** 10.3389/fpls.2022.907839

**Published:** 2022-05-30

**Authors:** Rihan Da, Minhui Hao, Xuetao Qiao, Chunyu Zhang, Xiuhai Zhao

**Affiliations:** Research Center of Forest Management Engineering of State Forestry and Grassland Administration, Beijing Forestry University, Beijing, China

**Keywords:** community functional composition, environmental drivers, functional trait, intraspecific variation, spatial scale, temperate forest

## Abstract

Understanding the trait–environment relationships has been a core ecological research topic in the face of global climate change. However, the strength of trait–environment relationships at the local and regional scales in temperate forests remains poorly known. In this study, we investigated the local and regional scale forest plots of the natural broad-leaved temperate forest in northeastern China, to assess what extent community-level trait composition depends on environmental drivers across spatial scales. We measured five key functional traits (leaf area, specific leaf area, leaf carbon content, leaf nitrogen content, and wood density) of woody plant, and quantified functional compositions of communities by calculating the “specific” community-weighted mean (CWM) traits. The sum of squares decomposition method was used to quantify the relative contribution of intraspecific trait variation to total trait variation among communities. Multiple linear regression model was then used to explore the community-level trait–environment relationships. We found that (*i*) intraspecific trait variation contributed considerably to total trait variation and decreased with the spatial scale from local to regional; (*ii*) functional composition was mainly affected by soil and topography factors at the local scale and climate factor at the regional scale, while explaining that variance of environment factors were decreased with increasing spatial scale; and (*iii*) the main environment driver of functional composition was varied depending on the traits and spatial scale. This work is one of the few multi-scale analyses to investigate the environmental drivers of community functional compositions. The extent of intraspecific trait variation and the strength of trait–environment relationship showed consistent trends with increasing spatial scale. Our findings demonstrate the influence of environmental filtering on both local- and regional-scale temperate forest communities, and contribute to a comprehensive understanding of trait–environment relationships across spatial scales.

## Introduction

Trait-based ecology assumes that there are a series of traits that are functional and that link the environment to the performance (e.g., growth and survival) and ultimate fitness of the entire plant by variation in these traits (Cornelissen et al., 2003; Violle et al., 2007; Schmitz et al., 2015). Many ecological issues can be effectively addressed with the deepening understanding of the plant functional traits (Kraft et al., 2008; Diaz et al., 2016). However, trait-based ecology measures the properties of individuals, thus it must be scaled to community and ecosystems to predict their dynamics and functioning (Enquist et al., 2015). Additionally, it can be better explain the variation in multiple ecosystem functions by focusing on the shape and shifts of trait distributions in communities (Savage et al., 2007; Enquist et al., 2015). Therefore, examining how the functional composition of communities changes with environmental conditions is the key to understanding the role of persistent environment changes in driving community structure and ecosystem processes (Lavorel and Garnier, 2002; Funk et al., 2017; Wieczynski et al., 2019).

The community functional composition has been widely characterized by community-weighted mean (CWM) trait value (Funk et al., 2017). The CWM trait value corresponds to the average value of the functional trait of all individuals within community, which is typically calculated by multiplying species mean trait values by their relative abundances (Garnier et al., 2004; Violle et al., 2007). However, a limitation of this approach is that it ignores the contribution of intraspecific trait variation. The justification of using species mean trait value is based on the assumption that interspecific variation accounts for more fraction of the total trait variability as compared to intraspecific variation (Garnier et al., 2001). While abiotic filters act on individuals rather than species, and consequently relying on species mean trait values may produce misleading or spurious conclusions (Violle et al., 2012). It is becoming increasingly clear that intraspecific variation contributes considerably to trait variation and can influence community composition and dynamics (Messier et al., 2010; Westerband et al., 2021). Recent studies demonstrate that intraspecific variation had large effects on overall functional composition (Pichon et al., 2022). Therefore, incorporating both inter- and intraspecific trait variation when calculating community-weighted metrics will provide a reasonable approximation of community functional composition.

Intraspecific trait variation results from genotypic variation, phenotypic plasticity, or their interaction (Schlichting, 1986; Scheiner, 1993). Consequently, the intraspecific trait variation is influenced by the extent of gene flow and the degree of environmental heterogeneity (Bolnick et al., 2003; Baythavong, 2011). Increasing spatial scale is typically associated with increasing environmental heterogeneity and hence with increasing trait variation (Messier et al., 2016). The “spatial variance partitioning” (SVP) hypothesis proposes that intraspecific and interspecific trait variation are both low at small scales and increases with spatial scale, saturating at different points along the continuum (Albert et al., 2011). However, intraspecific trait variation plateaus when the scale encompasses the entire species’ distribution (Siefert et al., 2015), and thus the relative contribution of intraspecific trait variation to total trait variation among communities is expected to decrease with increasing spatial extent from local to regional and global scales (Albert et al., 2011). Although the extent of intraspecific trait variation across levels of biological organization has been widely reported (Westerband et al., 2021), whether and how it varies with spatial scales in temperate forest communities remains unclear. Therefore, it is necessary to quantify the contributions of intraspecific variation on total trait variation across spatial scales.

Geographical variation of functional traits is closely related to environmental conditions. Environmental filtering hypothesis proposed that the abiotic environment factors select species from the regional pool with similar trait values into communities to adapt to the local environment conditions (Keddy, 1992; Kraft and Ackerly, 2010). Assessing trait–environment relationships across spatial scales can aid in advancing our ability to use traits to predict the composition of communities in response to novel environmental conditions. Trait–environment relationships at the global scale have been gradually unraveled in previous studies using global plant trait databases (Bruelheide et al., 2018; Wieczynski et al., 2019). Nevertheless, these large-scale trait patterns were failed to account for ecological processes operating at local scales that govern the functional profile of community assemblages (Pinho et al., 2021). The functional composition of communities sampled at fine-grained plots was the direct outcome of the interaction both local and large-scale factors (Bruelheide et al., 2018). For instance, communities across Neotropical moist forests share similar sets of functional strategies driven by current climatic conditions (Pinho et al., 2021). However, it remains unknown to what extent community-level trait composition depends on regional-scale environmental drivers, particularly the effects of local-scale environmental factors (e.g., soil and topography factors) in temperate forests.

Temperate forests cover around 16% of the global forest area and 34% of global carbon sinks (Hansen et al., 2010; Pan et al., 2011). These forests are more altered, fragmented, and reduced than most other forest ecosystems due to their locations in the most densely populated areas of the world (Landuyt et al., 2019). Given that temperate forests are facing the risks of biodiversity loss in the context of global climate change (Loarie et al., 2009), an urgent concern of ecological research is to deepen the understanding of how their functional composition responds to environmental drivers. Generally, the functional composition of communities was mainly affected by climate factors at the regional scale (Zhou et al., 2013; Pinho et al., 2021), while soil and topography factors at the local scale (Shen et al., 2016; Libalah et al., 2017). However, the relative importance of these environmental factors, as well as the strongest environmental predictor of temperate forest communities remains less clear. Thereby, quantifying and assessing environmental drivers of functional composition across local and regional scales is essential for guiding land policy and management decisions for temperate forests.

In this study, we calculated a “specific” CWM trait value, which combines both inter- and intraspecific trait variation to assess the trait–environment relationships. Our dataset consisted of large region forest plots and local-scale permanent forest observation study site of the natural broad-leaved temperate forest distributed throughout northeastern China. We focused on the two spatial scales (local and regional), and first quantified the contribution of intraspecific variation to the total among-community variation of different functional traits. Then we assessed how environmental conditions influence the trait composition of forest communities. Finally, we determined the strongest environment predictors of community functional compositions. Our study had two principal objectives: (*i*) to test the contribution of intraspecific variation on total trait variation and how it changes with spatial scale; and (*ii*) to test the effects of environmental filtering on the functional composition of forest communities across different spatial scales. We anticipated that the extent of intraspecific trait variation and the strength of trait–environment relationship would both decrease with increasing spatial scale.

## Materials and Methods

### Study Area

Our study was conducted in natural broad-leaved temperate forests of northeastern China. The study region covers seven mountain ranges: the Longgang, the Hadaling, the Changbai, the Zhangguangcailing, the Laoyeling, the Wanda, and the Lesser Khingan Mountain areas. The area is characterized by a temperate continental climate affected by monsoon, with four distinct seasons. The annual average temperature ranges from −0.4 to 10.4°C, and the annual precipitation ranges from 368 to 879 mm.

**Figure 1:**
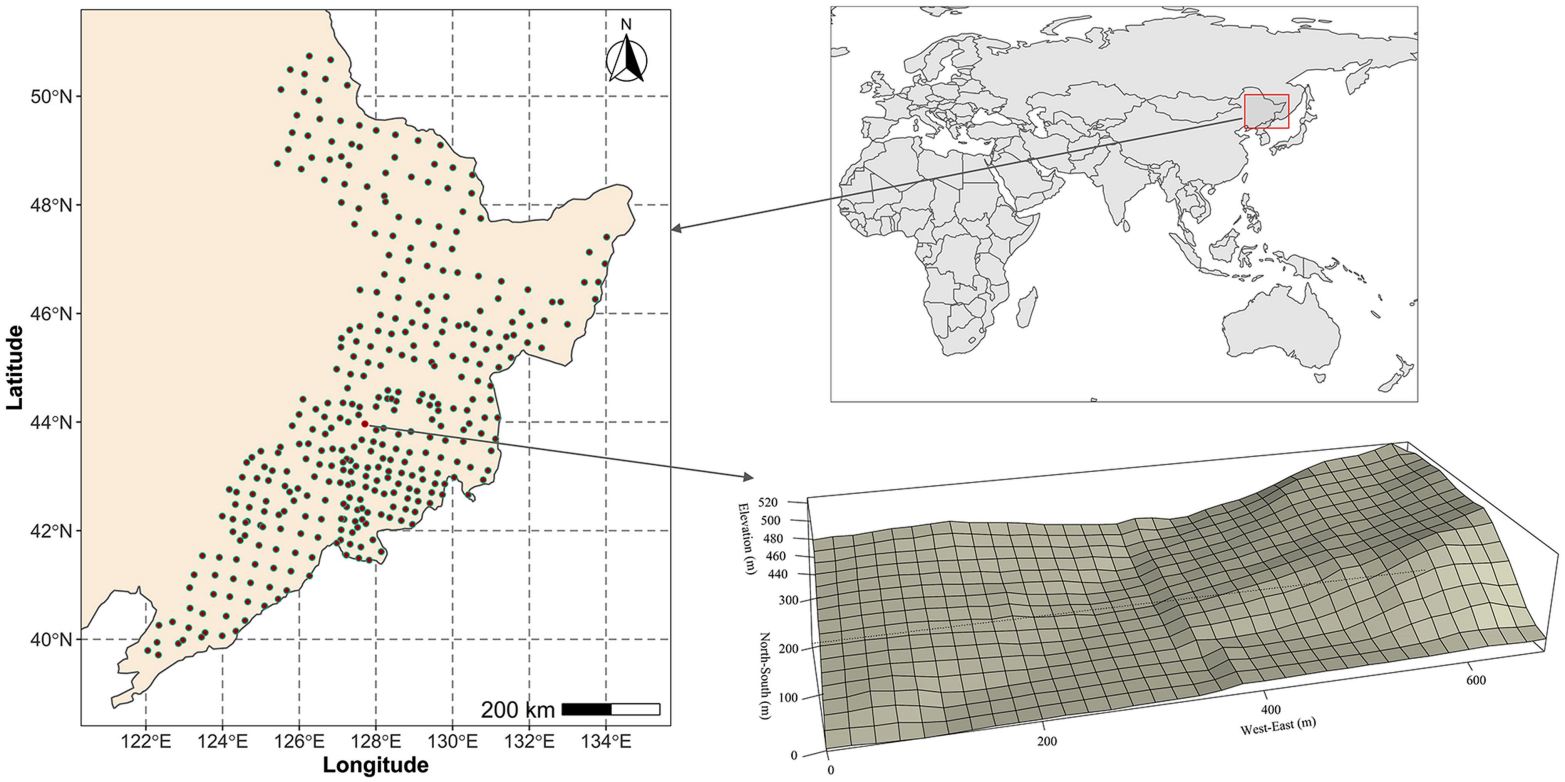
Spatial distribution of the surveyed plots.

For the regional-scale study, we established a total of 358 circular forest plots (0.1 ha) that cover the whole study region in the summer of 2017 and 2018 (Figure 1; Zhang et al., 2020; Qiao et al., 2021). The latitudinal and longitudinal, respectively, ranges extend from 39°42′48″ to 50°44′22″ and from 122°02′57″ to 133°58′35″. In addition, the site located in the middle of the regional study area were used for the local-scale study (43°57′54″–43°58′16″N, 127°42′47″–127°43′19″E; Figure 1). A permanent forest observation study covering an area of 21.12 ha (660 × 320 m) was established in the summer of 2009 (Figure 1; Zhang et al., 2012; Hao et al., 2018). This was then subdivided into a total of 528 square plots (20 × 20 m).

All individual woody trees with a diameter at breast height (DBH) > 5 cm were identified at the species level and measured in each plot both for the regional and local scales. In these plots, a total of 17,239 individuals of 21 species and 27,925 individuals of 58 species were recorded (Supplementary Table S1).

### Environment Variables

For the regional-scale study, a total of 15 environment variables were obtained, then subdivided into climate, soil, and topography (see more details in Supplementary Table S2). Climate variables, including mean annual temperature, mean diurnal range, max temperature of warmest month, mean annual precipitation, precipitation seasonality, precipitation as snow, and solar radiation, were extracted from WorldClim 2 and the ClimateAP Dataset (Fick and Hijmans, 2017; Wang et al., 2017). Most of the soil variables, that is, coarse fragments, volumetric water content, total nitrogen content, C/N ratio, and pH in water, were calculated from the WISE30secv.1 (Batjes, 2015). The one exception was soil depth, which was measured in the field. Two topography variables (elevation and slope) used for this study were assessed by our own field observations.

As for the local-scale study, four topography and eight soil variables were estimated as proxies of the environmental conditions (Supplementary Table S3). The topography variables, including elevation, convexity, aspect, and slope, were measured and calculated for each 20 × 20 m plot (Zhang et al., 2012; Hao et al., 2020). Since the aspect is a circular variable, we using a method (Zhong et al., 2021) standardized it to reflect northerly (COSA) and easterly (SINA) facing slopes (range = − 1 to + 1). These two variables, respectively, increase with the more northerly and easterly directions of aspect. Eight soil properties, namely, organic carbon mass content, total nitrogen, available nitrogen, total phosphorus, available phosphorus, total potassium, available potassium, and soil acidity, were measured from soil samples that extracted to a depth of 10 cm in each 40 × 40 m plot. These soil variables were then interpolated to grids of 20 × 20 m using the Ordinary Kriging in the *gstat* package.

### Trait Data

In this study, we focused on the key functional traits that are believed to influence plant performance and reflect its life-history strategies (Diaz et al., 2016). Four-leaf traits and one stem trait, including leaf area (LA), specific leaf area (SLA), leaf carbon content (LCC), leaf nitrogen content (LNC), and wood density (WD), were measured in 2017 and 2018. More details of these traits and their significance can be seen in Table 1. We sampled one individual for each particular species that occurred in each plot, and they were available for both the regional and local-scale plots. We, respectively, sampled 3,803 and 2,516 individual trees from the local- and regional-scale plots. We also used random sampling instead of traditional sampling protocols to reveal the true extent of the intraspecific trait variations (Westerband et al., 2021). The specific species mean trait values were represented by the trait values of a given sampled individual. At least five fresh, intact, and fully expanded leaf samples were taken from each sampled individual on the highest part of the tree crown, which was exposed to direct sunlight or high lateral light levels (Liu et al., 2013). Wood core samples were extracted from the tree trunk at a height of 1.3 m using an increment borer (5 mm, Suunto, Finland). To not affect the next forest censuses of this permanent forest observation study site used in the local-scale study, we only extracted wood cores within the regional-scale plots. For this reason, subsequent analyses for relationship between wood density and environmental conditions were conducted only at the regional scale and reported the results only in Supporting Information. LA and SLA (leaf area/dry matter) were obtained using the standard methods (Cornelissen et al., 2003). Leaf elements (LCC and LNC) were gathered using elemental analyzer PE2400 SeriesII (PerkinElmer Inc., United States) and J200 Tandem LA/LIBS system (Applied Spectra Inc., Fremont, CA, United States). WD was calculated as the ratio of the wood core dry mass (80°C, 72 h) to its fresh volume (Williamson and Wiemann, 2010; Hao et al., 2018).

**Table 1:** Significance of functional traits used in this study.

Functional traits	Abbreviation	Units	Functional significance
Leaf area	LA	mm^2^	Light acquisition
Specific leaf area	SLA	mm^2^/g	Leaf economic spectrum; light interception efficiency; plant shade tolerance
Leaf carbon content	LCC	%	Carbon assimilation rate
Leaf nitrogen content	LNC	%	Leaf economic spectrum; photosynthetic capacity; metabolic rate
Wood density	WD	g/cm^3^	Wood economic spectrum; volume growth; stem defense

### Statistical Analysis

We calculated the CWM for each trait within each community using the following equation (Garnier et al., 2004; Wieczynski et al., 2019):


$${\mathrm{CWM}}_{j,k}=\overset{n_{k}}{\underset{i}{\sum }}P_{i,k}T_{i,j,k}$$

Where $n_{k}$ and $P_{i,k}$ are the total number of species and relative abundance of species i in plot k, respectively; and $T_{i,j,k}$ is the mean value of trait j for species i in plot k, meaning that each species has a specific trait value within each plot, rather than a uniform species mean trait value. Then we log-transformed the size-dependent trait (leaf area) prior to calculation to eliminate bias (Kerkhoff and Enquist, 2009). This CWM computation was performed for each plot at the local and regional scales.

To quantify the relative contribution of intraspecific trait variation on total among-community trait variance, we followed the sum of squares decomposition method proposed by Leps et al. (2011). This method consists of following steps. First, for each trait and plot, we computed three types of CWMs. (1) CWM_specific_ was calculated following the previously described equation, which includes both intraspecific trait variation and species turnover effects. (2) The CWM_fixed_ was calculated using species mean trait values averaged across all plots, including only the effects of species turnover. (3) The intraspecific variability effect was then measured as CWM_intra_ = CWM_specific_ - CWM_fixed_ (Siefert et al., 2015). Second, the total sum of squares (SS_specific_) of the community-level trait variation was decomposed into “fixed” (SS_fixed_), “intraspecific” (SS_intra_), and “covariation” (SS_cov_) components (i.e., SS_specific_ = SS_fixed_ + SS_intra_ + SS_cov_). Lastly, the relative contribution of intraspecific trait variation was assessed as the ratio between the regression sum of squares of the intraspecific trait variation model (SS_intra_) and the total sum of squares of the model including both components (SS_specific_). Here we focused on the relative rather than the absolute extent of intraspecific trait variation, because it allows comparison of multiple traits measured in different units or on different scales (Siefert et al., 2015).

The multiple linear regression models were then used to assess the effect of environment variables on community functional composition. Before fitting these models, all predictors and response variables were standardized to interpret the relative importance of the environment variables on a comparable scale (Gross et al., 2017). For each model, the variance inflation factors (VIF) of all predictors were less than 10, so as to avoid multicollinearity. Then, a model selection procedure based on minimizing Akaike’s information criterion (AIC, ∆AIC < 2) was used to select the best predictors of community functional trait composition (Burnham and Anderson, 2002). The *dredge* function in the *MuMIn* package was used for this procedure (Bartoń, 2014). The relative importance of the different environmental factors was then evaluated using a method similar to a variance decomposition analysis (Gross et al., 2017; Le Bagousse-Pinguet et al., 2017). The relative effects of each environmental factor were calculated as the ratio of the sum of its parameter estimate to the sum of all parameter estimates in the model, expressed as a percentage. All statistical analyses were performed using R 4.0.5 (R Core Team, 2021) for both the local and regional scales.

## Results

### Intraspecific Trait Variation Across Spatial Scales

**Figure 2:**
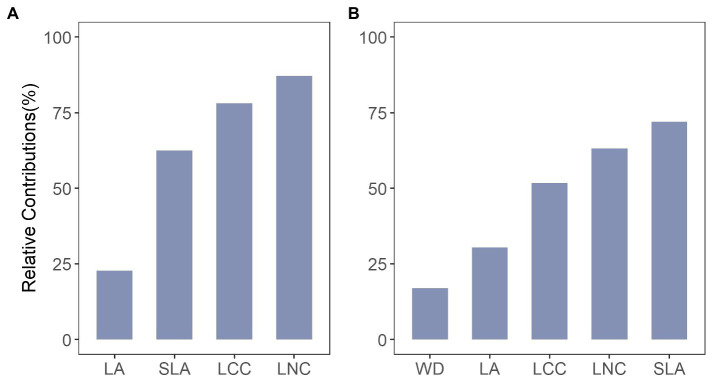
Relative contributions of intraspecific trait variation to explaining variability in total trait variation for traits at **(A)** local scale and **(B)** regional scale. LA, leaf area; LCC, leaf carbon content; LNC, leaf nitrogen content; SLA, specific leaf area; WD, wood density.

The results showed that intraspecific trait variation contributed 23–87% of the total among-community trait variation for the local scale and 17–72% for the regional scale (Figure 2). The relative importance of intraspecific variability differed among traits and spatial scales. At the local scale (Figure 2A), a relatively large portion of the total variance was attributed to intraspecific trait variation for leaf chemical traits (LCC and LNC), compared to leaf morphological traits (LA and SLA). At the regional scale, the intraspecific variance was highest for SLA (72%), and lowest for wood density (WD), which had only 17% of the total trait variation being attributed to intraspecific trait variation (Figure 2B). We even observed that intraspecific trait variation was equivalent to or surpassed interspecific trait variation for most cases (six out of nine). Overall, intraspecific trait variation explained an average of 63% for the local scale and 54% for the regional scale of the total trait variation (only for the traits that exist in both scales).

### Explained Variance of Environmental Factors on Community Functional Composition

**Figure 3:**
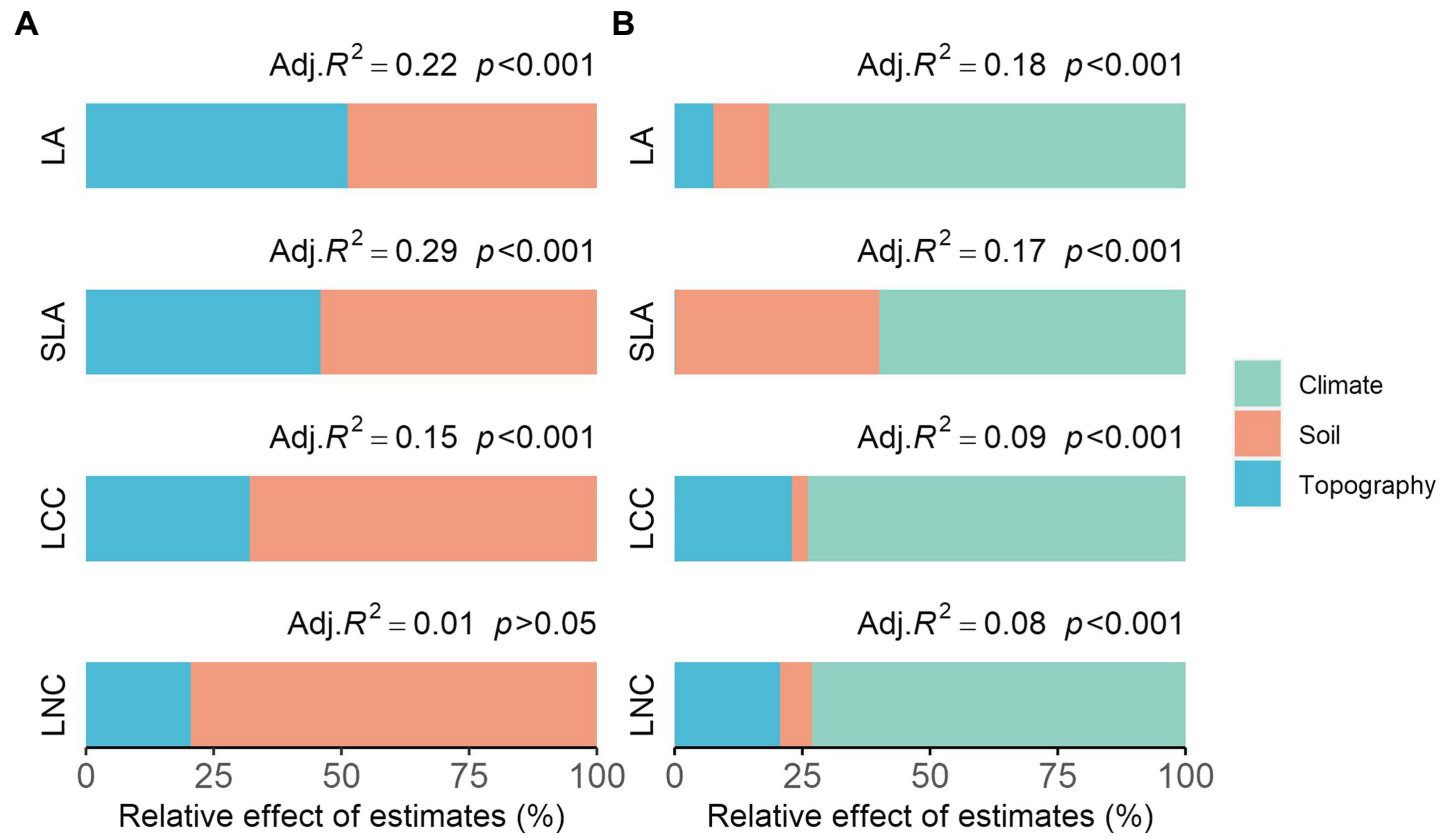
The relative importance of each environmental factor on community functional composition across the **(A)** local scale and **(B)** regional scale, expressed as the percentage of explained variance. We showed the adjusted *R*^2^ and the value of *p* of the averaged models. LA, leaf area; LCC, leaf carbon content; LNC, leaf nitrogen content; SLA, specific leaf area.

We observed different trait–environment patterns across the two spatial scales (Figure 3). The variance in community functional composition explained by each environmental factor and their relative importance was varied between functional traits (Figure 3). For the local scale, the predictive power of the models was greatest for the SLA (Figure 3A, adjusted *R*^2^ = 0.29), followed by LA (0.22), compared to LCC (0.15) and LNC (0.01; insignificant, *p* = 0.135). Soil factors were responsible for an average of 57% (49–68%) of the explained variance in community functional composition (not considering the insignificant model), which was greater than that explained by the topography factors (43%). For the regional scale, the predictive power of the models on community functional composition was greater for the LA (Figure 3B, adjusted *R*^2^ = 0.18), and also reached a similar level for the SLA (0.17) compared to LCC (0.09) and LNC (0.08). Climate factors (60–82%) played a dominant role in determining the functional composition of the regional scale, followed by soil (3–40%) and finally topography (0–23%). It is also noteworthy that when applied to wood density (WD), the model showed the greatest predictive power compared to the other traits on the regional scale (Supplementary Figure S1, adjusted *R*^2^ = 0.25), which was also mainly explained by climate factors (70%). The predictive power of our models was decreased when explaining larger spatial scale trait–environment patterns (three out of four traits; Figure 3).

### Environment Predictors of Functional Composition

**Figure 4:**
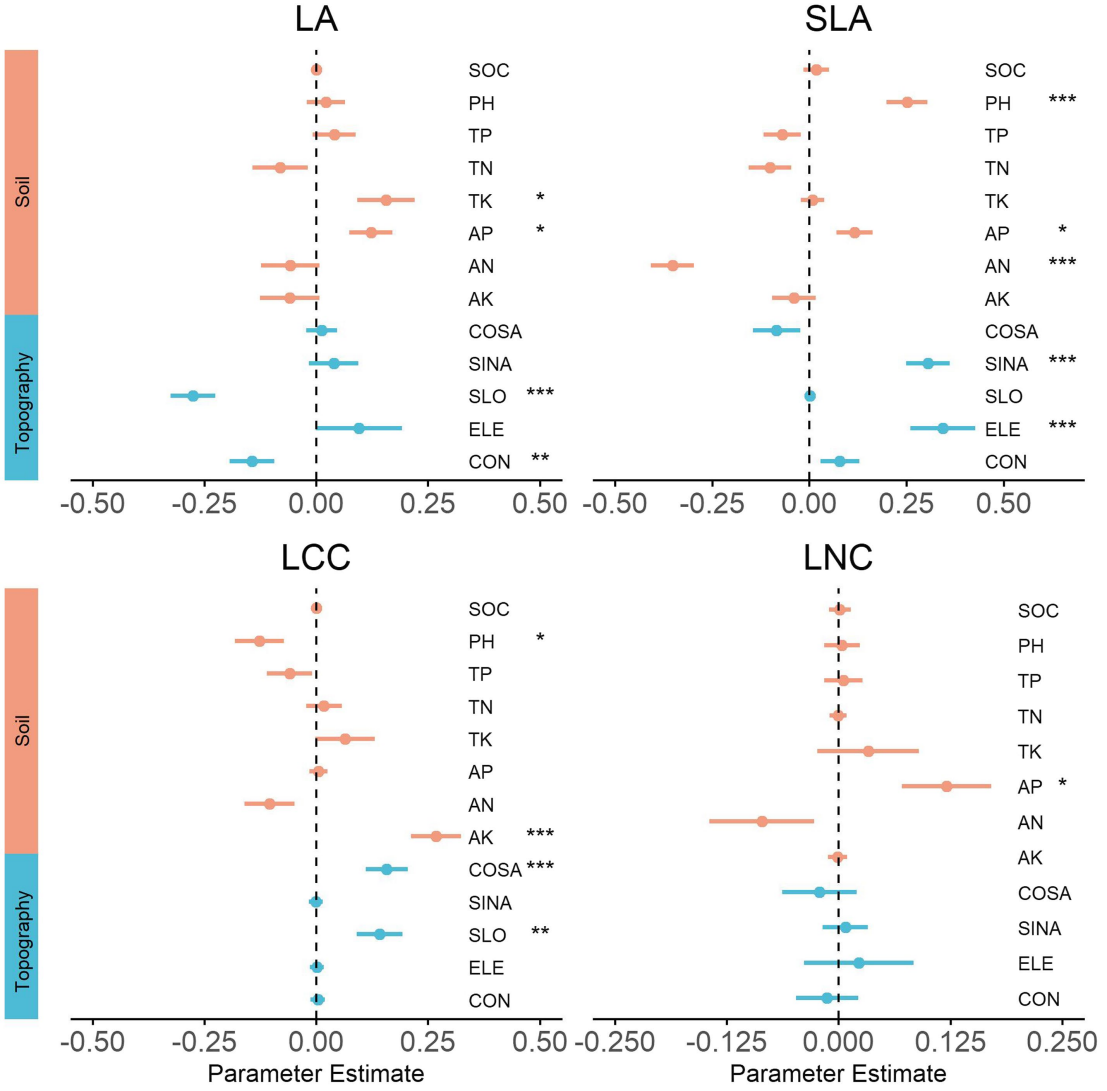
Effects of environmental variables on community functional composition for the local scale. We show the averaged parameter estimates (standardized regression coefficients) of model predictors and the associated 95% confidence intervals. The *p*-value of each predictor are given as: (.), *p* < 0.1; ^*^p < 0.05; ^**^p > 0.01; ^***^p < 0.001. SOC, organic carbon mass content; PH, soil acidity; TP, total phosphorus; TN, total nitrogen; TK, total potassium; AP, available phosphorus; AN, available nitrogen; AK, available potassium; COSA, North aspect; SINA, East aspect; SLO, slope; ELE, elevation; CON, convexity.

At the local scale, the strongest individual environmental driver of each community functional composition varied widely among the traits (Figure 4). Specifically, slope (SLO) was the main driver and had a significant negative relation with LA. Available nitrogen (AN) and elevation (ELE) both had significantly negative and positive relationships with SLA. Available potassium (AK) was the strongest predictor of LCC, which explained up to 28% of the total variance.

**Figure 5:**
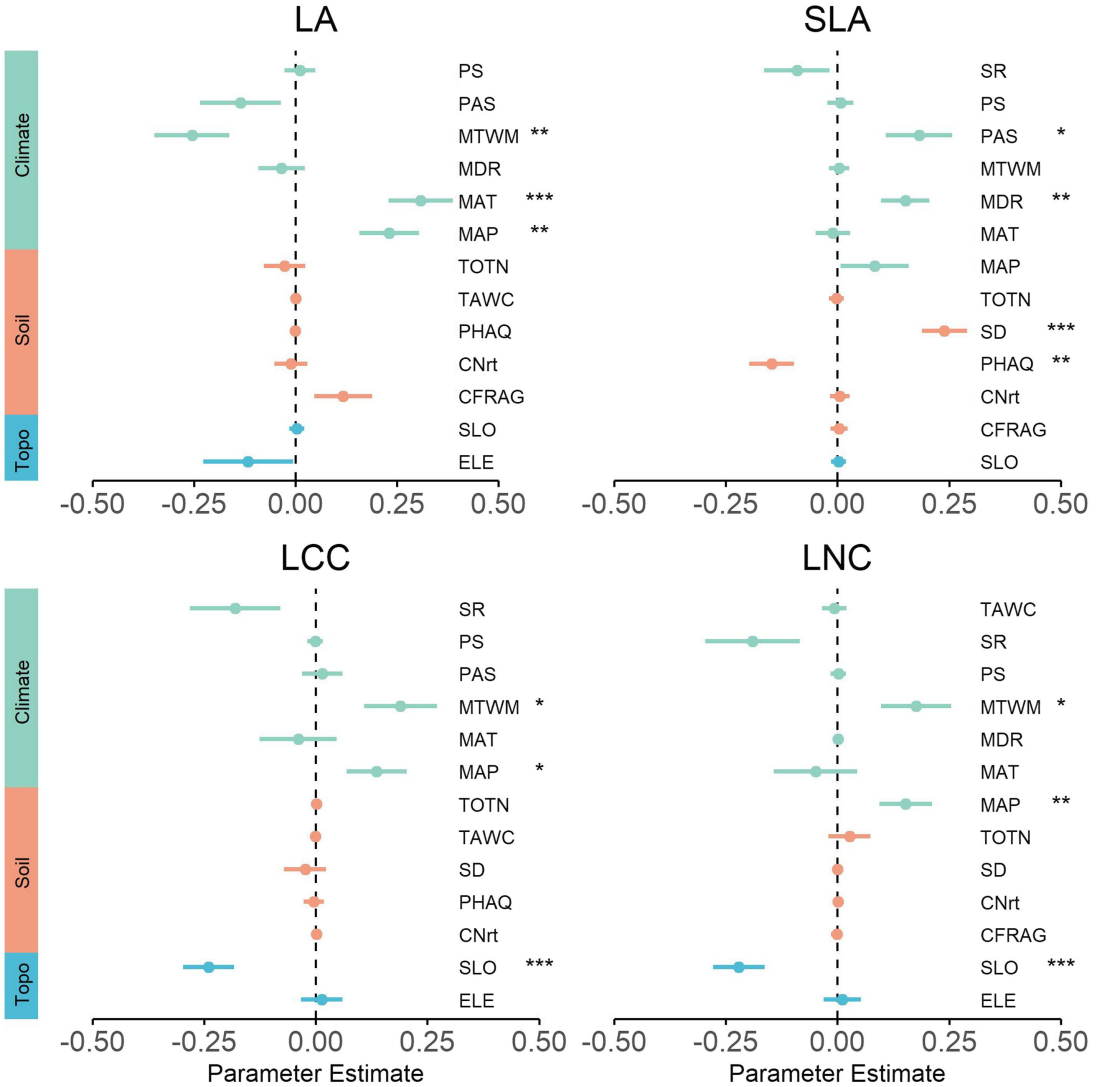
Effects of environmental variables on community functional composition for the regional scale. We show the averaged parameter estimates (standardized regression coefficients) of model predictors and the associated 95% confidence intervals. The *p*-value of each predictor are given as: (.), *p* < 0.1; ^*^p < 0.05; ^**^p > 0.01; ^***^p < 0.001. MAT, mean annual temperature; MDR, mean diurnal range; MTWM, max temperature of warmest month; MAP, mean annual precipitation; PS, precipitation seasonality; PAS, precipitation as snow; SR, solar radiation; CFRAG, coarse fragments; TAWC, volumetric water content; TOTN, total nitrogen content; CNrt, C/N ratio; PHAQ, pH in water; SD, soil depth; ELE, elevation; SLO, slope.

At the regional scale, mean annual precipitation (MAP) and mean annual temperature (MAT) were among the strongest predictors in most traits, signifying the influence of these two climatic factors on community functional composition (Figure 5; Supplementary Figure S1). However, SLA was weakly related to precipitation and temperature, being mainly driven by soil factors (soil depth and pH). Several different climate factors (MTWM, MDR, and PAS) also exhibited significant relations with trait composition. LCC and LNC exhibited very similar trends between community functional composition and topography factor, with values that were strongly negatively related to slope (SLO). In contrast, WD was strongly positively related to slope (SLO; Supplementary Figure S1). Interestingly, elevation (ELE) was determined to be insignificantly correlated with the trait in all cases.

## Discussion

### Intraspecific Trait Variation Differed Among Traits and Spatial Scales

We first explored how greatly intraspecific trait variation is found among communities in the local- and regional-scale temperate forests. Community-level trait variations were mainly dominated by intraspecific trait variation (i.e., explained an average of 63 and 54% for the local- and regional-scale, respectively). The results were similar to previous studies in that intraspecific trait variation consistently accounted for a significant proportion of the total trait variation (Messier et al., 2010; Siefert et al., 2015; Anderegg et al., 2018). Our results highlight the contribution of intraspecific variation on the total trait variation, thus we suggest considering it when assessing the trait–environment relationships. There is one enlightening result we found in our study, namely, that the relative contribution of intraspecific trait variation was decreased with spatial scale from local to regional for leaf chemical traits (LCC and LNC). This finding supports the SVP hypothesis, which lead to general predictions that the reduction in the extent of intraspecific trait variation at larger scales, and higher interspecific trait variation than intraspecific trait variation at larger scales (Albert et al., 2011). In contrast, we found that the relative contribution of intraspecific trait variation was increased with increasing spatial scale for leaf morphological traits (SLA and LA). Therefore, the predictions of SVP hypothesis do not hold in some cases, because whether intraspecific trait variation will exceed interspecific trait variation depends on species distributions, gene flow, environmental heterogeneity, and the traits of interest (Westerband et al., 2021).

We also observed considerable differences in the amount of intraspecific trait variance depending on the different functional traits. Specifically, the most used leaf trait in ecological analyses, SLA, showed the higher variation explained by intraspecific trait variance (62% for local and 72% for regional scale). Meanwhile, LA showed the lowest intraspecific trait variance and the nutrient concentration (LCC and LNC) were more similarly distributed at both local and regional scales. For the wood density of the regional scale, intraspecific variation contributed only 17% of the total trait variation. These results were consistent with those of previous studies (Albert et al., 2010; Kichenin et al., 2013; Kang et al., 2014; Messier et al., 2016), and these different extents of trait variance were likely to reflect varying degrees of phenotypic plasticity and genetic regulation (Westerband et al., 2021). However, it should be noted that measurement errors also contributed to apparent intraspecific variation, and not all trait variation was due to environmental acclimation. In addition, this study only focused on the broad-leaved temperate forests in this study, thus more research is required regarding coniferous forests, and even coniferous and broad-leaved mixed forests, so as to explore a more comprehensive result on intraspecific trait variation.

### Trait–Environment Patterns Differ Across Spatial Scales

Understanding the relative effects of environmental factors on community functional composition across different spatial scales remains an important challenge. In this study, we found that trait–environment patterns differ across traits and spatial scales. Soil factors explained more variation in community functional composition than did topography at the local scale for traits linked to the leaf economics spectrum (SLA and LCC). This result was consistent with those of previous studies in that the traits related to resource investment trade-offs were shifts in response to soil fertility gradient at the local-scale (Cornwell and Ackerly, 2009; Libalah et al., 2017). However, the relative importance of topography factors found may be equivalent to soil factors for LA, which represent the light acquisition ability. This is due to the fact that topography, as an important driver, not only affected soil moisture and nutrient availability (John Chandran et al., 2007), but also influenced the trees’ interception for light (Shen et al., 2016). Unexpectedly, the association of LNC (which was another trait of the leaf economics spectrum) to environmental factors was insignificant, given that it has rarely been reported in previous studies. This insignificant relationship may demonstrate that in temperate broad-leaved forests, local environmental conditions are not the driving factor of leaf nitrogen content. As for the regional scale, climate factors (60–82%) played a dominant role in determining the functional composition of communities, which was consistent with the findings of studies regarding temperate moist forests and subtropical evergreen broad-leaved forests (Swenson and Weiser, 2010; Huang et al., 2021). The variance explained by soil factor was relatively high for SLA compared to other traits, proving that it was also one of the important drivers of specific leaf area at a larger scale. We note that the strongest community-level correlations with environment factors were found for WD, reflecting the trade-off between stem traits (wood economics spectrum), also exist at the regional scale.

Together, the strong trait–environment correlations provided evidence to support the environmental filtering hypothesis at both spatial scales (particularly at the local scale). However, we found that the explained variance of environment factors was decreased with increasing spatial scale (three out of four traits). This result signifies that (*i*) the local abiotic forces were the main driver of functional trait composition of small-scale forest communities and that (*ii*) in addition to environmental factors, other factors (e.g., biotic interactions and genetic variability) also played an important role in driving regional-scale community functional composition. Overall, the environment represented the important factor shaping the functional composition of local and regional temperate forest communities, thus highlighting environment variability as potentially being the primary driver of this variation. Future work must use trait-based models based on trait–environment relationships and integrate biotic interactions to predict changes in community composition with accurate and consistent environmental datasets.

### Predictors of Community Functional Composition Across Different Scales

Our study results showed that the main environment predictor of each community functional composition was varied depending on the traits and spatial scale. For local-scale communities, the soil properties, including total potassium, available nitrogen, available phosphorus, available potassium, and soil acidity (pH), were significantly related to different trait compositions. This demonstrates that soil fertility also determined a shift in functional traits composition of communities at the local scale, which was consistent with the result of a previous study performed in a tropical rainforest (Libalah et al., 2017). In addition, slope was the main driver of LA and had a significant negative relation, which even had a significant positive effect on LCC. Elevation was the strongest predictor of SLA, explaining up to 19% of the total variance. Aspect (including SINA and COSA) had a strong effect on SLA and LCC, reflecting the light and water availability changes with the topography conditions (elevation, slope, aspect, and convexity), and resulting in different combinations of functional trait composition of the plant life-history strategies (Yao et al., 2021). Together, our findings provided evidence that local conditions (soil and topography) strongly affected the trait dissimilarity of forest communities.

As for the regional-scale communities, we observed that mean annual precipitation and temperature were among the strongest predictors in most of the measured traits, thus highlighting the influence of these two climatic factors on community functional composition, which was consistent with the result of previous studies performed at both regional and global scales (Maire et al., 2015; Muscarella and Uriarte, 2016; Chelli et al., 2019). While other climate factors, such as snow (PAS), have been reported as the influential abiotic variable affecting functional composition, in this study the same relation with SLA was also found to be present (Happonen et al., 2019). In addition, it is noteworthy that soil depth exhibited the strongest correlations with SLA, which is in line with previous research showing that communities revealed a gradual change in resource-use strategies from shallow soils to the deeper and more fertile soils (Bernard-Verdier et al., 2012; Gianasi et al., 2020). Furthermore, we noted extremely significant correlations between slope and traits related to resource utilization strategy (LCC, LNC, and WD). These relations were likely due to the local changes in habitat (especially the thermal conditions) induced by slope changes (Auslander et al., 2003). This demonstrates that slope can be a prominent topography factor for predicting shifts in functional composition in both local- and regional-scale communities. Unexpectedly, the insignificant relationships between elevation and traits contrasted with previous work in which communities exhibited significant and substantial shifts in trait composition across elevation gradient (Hulshof et al., 2013; Wieczynski et al., 2019). This observation may provide evidence that elevation is not the driver of trait composition of natural broad-leaved temperate forests.

## Conclusion

Assessing the trait–environment relationships across spatial scales is necessary for biodiversity conservation and vegetation restoration of forest communities. This study is one of the few multi-scale analyses to investigate environmental drivers of community functional composition in temperate forests. We first explored the extent of trait variation and found that intraspecific trait variation contributed considerably to total trait variation, which was decreased with the increasing spatial scale. Our results highlighted the importance of intraspecific variation on total trait variation and suggested that link it to the higher-order ecological processes. Next, we observed different trait–environment patterns across different spatial scales. Soil and topography factors acted together in determining the functional composition of communities at the local scale, while climate factor was the dominant driver at the regional scale. However, we also found that the explained variance of environment factors decreased with increasing spatial scale. Finally, we found the strongest environment predictor of community functional composition was varied depending on the traits and spatial scale. In conclusion, our research performed in this study not only contributes to a comprehensive understanding of trait–environment relationships across spatial scales, but also raises an important consideration for designing future trait-based studies.

## Data Availability Statement

The original contributions presented in the study are included in the article/Supplementary Material, further inquiries can be directed to the corresponding author.

## Author Contributions

RD and CZ conceived the idea of this study. MH, CZ, and XZ collected and extracted the data. RD analyzed the data and wrote the first draft of the manuscript. XQ, MH, CZ, and XZ contributed to writing *via* multiple rounds of revision. All authors contributed to the article and approved the submitted version.

## Funding

This study was supported by the Program of National Natural Science Foundation of China (31971650), the Key Project of National Key Research and Development Plan (2017YFC0504104), and Beijing Forestry University Outstanding Young Talent Cultivation Project (2019JQ03001).

## Conflict of Interest

The authors declare that the research was conducted in the absence of any commercial or financial relationships that could be construed as a potential conflict of interest.

## Publisher’s Note

All claims expressed in this article are solely those of the authors and do not necessarily represent those of their affiliated organizations, or those of the publisher, the editors and the reviewers. Any product that may be evaluated in this article, or claim that may be made by its manufacturer, is not guaranteed or endorsed by the publisher.
