# Supplementary material for: Unravelling Trait–Environment Relationships at Local and Regional Scales in Temperate Forests
Source: Front Plant Sci. 2022 May 30;13:907839. doi: 10.3389/fpls.2022.907839 (PMC9189410; doi:10.3389/fpls.2022.907839)
Supplement: Supplementary file 1 [file Data_Sheet_1.docx]

**SUPPORTING INFORMATION**

**Table S1** Species list shows the presence (1) and absence (0) of the species in the different spatial scale. Based on the APG Ⅲ classification system.

| **Species** | **Genus** | **Family** | **Local** | **Regional** |
| --- | --- | --- | --- | --- |
| *Acer mandshuricum* | *Acer* | Aceraceae | 1 | 1 |
| *Acer ginnala* | *Acer* | Aceraceae | 0 | 1 |
| *Acer barbinerve* | *Acer* | Aceraceae | 0 | 1 |
| *Acer ukurunduense* | *Acer* | Aceraceae | 0 | 1 |
| *Acer pseudosieboldianum* | *Acer* | Aceraceae | 0 | 1 |
| *Acer triflorum* | *Acer* | Aceraceae | 1 | 1 |
| *Acer tegmentosum* | *Acer* | Aceraceae | 1 | 1 |
| *Acer mono* | *Acer* | Aceraceae | 1 | 1 |
| *Toxicodendron vernicifluum* | *Toxicodendron* | Anacardiaceae | 0 | 1 |
| *Kalopanax septemlobus* | *Kalopanax* | Araliaceae | 0 | 1 |
| *Aralia elata* | *Aralia* | Araliaceae | 0 | 1 |
| *Betula platyphylla* | *Betula* | Betulaceae | 1 | 1 |
| *Betula costata* | *Betula* | Betulaceae | 1 | 1 |
| *Betula dahurica* | *Betula* | Betulaceae | 0 | 1 |
| *Alnus sibirica* | *Alnus* | Betulaceae | 0 | 1 |
| *Carpinus cordata* | *Carpinus* | Betulaceae | 1 | 1 |
| *Bothrocaryum controversum* | *Bothrocaryum* | Cornaceae | 0 | 1 |
| *Castanea mollissima* | *Castanea* | Fagaceae | 0 | 1 |
| *Quercus aliena* | *Quercus* | Fagaceae | 0 | 1 |
| *Quercus dentata* | *Quercus* | Fagaceae | 0 | 1 |
| *Quercus wutaishanica* | *Quercus* | Fagaceae | 0 | 1 |
| *Quercus acutissima* | *Quercus* | Fagaceae | 0 | 1 |
| *Quercus mongolica* | *Quercus* | Fagaceae | 1 | 1 |
| *Quercus variabilis* | *Quercus* | Fagaceae | 0 | 1 |
| *Juglans mandshurica* | *Juglans* | Juglandaceae | 1 | 1 |
| *Maackia amurensis* | *Maackia* | Leguminosae | 1 | 1 |
| *Robinia pseudoacacia* | *Robinia* | Leguminosae | 0 | 1 |
| *Gleditsia japonica* | *Gleditsia* | Leguminosae | 0 | 1 |
| *Morus alba* | *Morus* | Moraceae | 0 | 1 |
| *Syringa reticulata* | *Syringa* | Oleaceae | 0 | 1 |
| *Fraxinus rhynchophylla* | *Fraxinus* | Oleaceae | 1 | 1 |
| *Fraxinus mandschurica* | *Fraxinus* | Oleaceae | 1 | 1 |
| *Rhamnus davurica* | *Rhamnus* | Rhamnaceae | 0 | 1 |
| *Padus maackii* | *Padus* | Rosaceae | 0 | 1 |
| *Padus racemosa* | *Padus* | Rosaceae | 0 | 1 |
| *Armeniaca mandshurica* | *Armeniaca* | Rosaceae | 0 | 1 |
| *Cerasus maximowiczii* | *Cerasus* | Rosaceae | 0 | 1 |
| *Crataegus maximowiczii* | *Crataegus* | Rosaceae | 0 | 1 |
| *Pyrus ussuriensis* | *Pyrus* | Rosaceae | 0 | 1 |
| *Malus baccata* | *Malus* | Rosaceae | 1 | 1 |
| *Cerasus serrulata* | *Cerasus* | Rosaceae | 0 | 1 |
| *Sorbus alnifolia* | *Sorbus* | Rosaceae | 1 | 1 |
| *Phellodendron amurense* | *Phellodendron* | Rutaceae | 1 | 1 |
| *Salix koreensis* | *Salix* | Salicaceae | 0 | 1 |
| *Salix raddeana* | *Salix* | Salicaceae | 0 | 1 |
| *Populus ussuriensis* | *Populus* | Salicaceae | 0 | 1 |
| *Salix taraikensis* | *Salix* | Salicaceae | 0 | 1 |
| *Salix matsudana* | *Salix* | Salicaceae | 0 | 1 |
| *Populus davidiana* | *Populus* | Salicaceae | 1 | 1 |
| *Populus koreana* | *Populus* | Salicaceae | 0 | 1 |
| *Styrax obassia* | *Styrax* | Styracaceae | 0 | 1 |
| *Tilia mandshurica* | *Tilia* | Tiliaceae | 1 | 1 |
| *Tilia amurensis* | *Tilia* | Tiliaceae | 1 | 1 |
| *Ulmus pumila* | *Ulmus* | Ulmaceae | 0 | 1 |
| *Ulmus davidiana* | *Ulmus* | Ulmaceae | 1 | 1 |
| *Ulmus macrocarpa* | *Ulmus* | Ulmaceae | 1 | 1 |
| *Celtis koraiensis* | *Celtis* | Ulmaceae | 0 | 1 |
| *Ulmus laciniata* | *Ulmus* | Ulmaceae | 1 | 1 |

**Table S2** Descriptive statistics of the climatic, soil, and topographic variables that used in regional-scale study.

| **Variables** | **Abbreviation** | **Unite** | **Mean** | **Minimum** | **Maximum** |
| --- | --- | --- | --- | --- | --- |
| **Climate** |  |  |  |  |  |
| Mean annual temperature | MAT | ℃ | 4.64 | -0.40 | 10.40 |
| Mean diurnal range | MDR | ℃ | 12.60 | 10.01 | 15.50 |
| Max temperature of warmest month | MTWM | ℃ | 28.62 | 22.60 | 31.50 |
| Mean annual precipitation | MAP | mm | 613.77 | 368.00 | 879.00 |
| Precipitation seasonality | PS | mm | 114.33 | 93.87 | 155.51 |
| Precipitation as snow | PAS | mm | 41.46 | 13.00 | 92.00 |
| Solar radiation | SR | kJ m^-2^ day^-1^ | 46.57 | 4.00 | 100.00 |
| **Soil** |  |  |  |  |  |
| Coarse fragments | CFRAG | % | 7.00 | 1.00 | 19.00 |
| Volumetric water content | TAWC | % | 16.53 | 15.00 | 21.00 |
| Total nitrogen content | TOTN | % | 1.86 | 0.63 | 4.60 |
| C/N ratio | CNrt | unitless | 11.37 | 8.00 | 14.00 |
| pH | PHAQ | unitless | 6.17 | 5.20 | 8.00 |
| Soil depth | SD | cm | 46.57 | 4.00 | 100.00 |
| **Topography** |  |  |  |  |  |
| Elevation | ELE | m | 408.20 | 79.00 | 1255.00 |
| Slope | SLO | degree | 14.90 | 0.00 | 47.00 |

**Table S3** Descriptive statistics of the soil and topographic variables that used in local-scale study.

| **Variables** | **Abbreviation** | **Unite** | **Mean** | **Minimum** | **Maximum** |
| --- | --- | --- | --- | --- | --- |
| **Soil** |  |  |  |  |  |
| Organic carbon mass content | SOC | g/kg | 62.08 | 29.36 | 132.90 |
| Total nitrogen | TN | g/kg | 5.01 | 2.01 | 18.75 |
| Available nitrogen | AN | mg/kg | 255.12 | 27.84 | 659.18 |
| Total phosphorus | TP | g/kg | 0.26 | 0.07 | 0.96 |
| Available phosphorus | AP | mg/kg | 16.59 | 6.64 | 35.71 |
| Total potassium | TK | g/kg | 4.03 | 3.49 | 4.40 |
| Available potassium | AK | mg/kg | 78.47 | 57.66 | 103.09 |
| Soil acidity | PH | unitless | 5.87 | 5.13 | 6.81 |
| **Topography** |  |  |  |  |  |
| Elevation | ELE | m | 459.87 | 426.32 | 519.69 |
| Convexity | CON | m | 0.01 | -6.62 | 4.70 |
| North aspect^1^ | COSA | unitless | -0.70 | -1.00 | 0.59 |
| East aspect^2^ | SINA | unitless | -0.30 | -1.00 | 0.98 |
| Slope | SLO | degree | 12.94 | 1.36 | 39.18 |

^1^cos((2π×aspect)/360); ^2^sin((2π×aspect)/360)


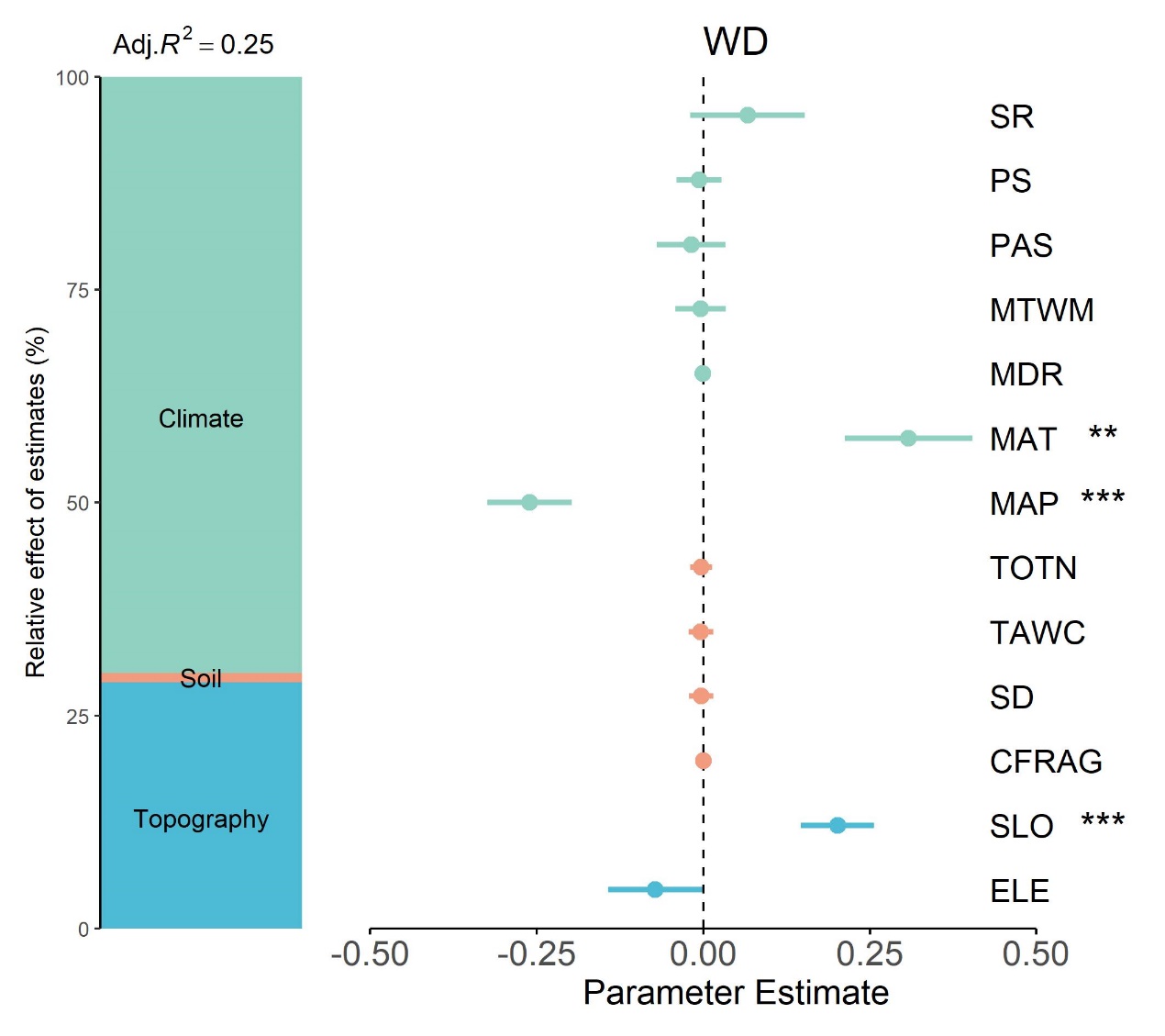


**Figure S1** Effects of environmental variables on community-weighted mean of wood density (WD). We show the averaged parameter estimates (standardized regression coefficients) of model predictors, the associated 95% confidence intervals and the relative importance of each environmental factor, expressed as the percentage of explained variance. The adj.*R*^2^ of the averaged models and the P-value of each predictor are given as: (.), *P* < 0.1; **P* < 0.05; ***P* > 0.01;****P* < 0.001.MAT, mean annual temperature; MDR, mean diurnal range; MTWM, max temperature of warmest month; MAP, mean annual precipitation; PS, precipitation seasonality; PAS, precipitation as snow; SR, solar radiation; CFRAG, coarse fragments; TAWC, volumetric water content; TOTN, total nitrogen content; SD, soil depth; ELE, elevation; SLO, slope.
